# Reply to Criticisms

**Published:** 1897-06

**Authors:** Louis Jack

**Affiliations:** President of International Dental Publishing Company


### Editorial.

### REPLY TO CRITICISMS.

THE American Academy of Dental Science recently very properly prepared a series of resolutions in denunciation of advertisements in dental journals which in their character are derogatory to professional honor, and tend to the degradation of professional spirit. These resolutions point to two kinds of advertisements. The one class relating to the solicitation of dentists to employ advertising agencies to prepare for them their notices of professional skill, in which it is maintained "that professional dignity and good advertising will work well together."

In the advertising pages of this journal there have unfortunately appeared for several issues the announcement by two of these writers of dental advertisements.

It is proper here to explain how this has occurred. Our advertising matter is under the management of an agent, who pursues his efforts in a simple business manner, and who could not be expected to have the fine ethical sense in the situation which should obtain. The member of the board, whose function it is to overlook such questions, had in his pressing daily duties neglected to scrutinize the pages until his attention was called to it by the resolutions above mentioned. The order has been given the agent to eliminate such advertisements in the future.

The other kind of advertisements alluded to in the resolutions are those concerning secret preparations of a dangerous character. Concerning these, the rule adopted by us to exclude them from the JOURNAL is that no such advertisement shall be accepted which is not accompanied by the formula, which must also be published as a part of the advertisement. Recently one of these has appeared in our pages where the contents of the preparation are given, but not the exact formula, which is not in strict conformity with the rule. This will not be again published.

We would be glad if the profession would sustain the application of the above rule to all secret preparations,—namely, amalgams, phosphatic cements, and similar preparations. The general profession certainly is at fault in the use of preparations where the formula of their composition is not publicly declared. It would be a great benefit if the public law required that all preparations connected with the treatment of disease were obliged to have stated on the package the exact formula, with a penalty attached to the avoidance of this salutary requirement. This, considering the character of our form of government, would appear to be impossible of execution, since it comes within the function of municipal law, relegated to the individual States. Therefore no concert of action upon this question is capable of being secured among the different States. It is obvious that any attempt by one State would be inoperative in any of the others. The interests of private concerns are continually acting against the application and enforcement of any measure so well calculated to prevent the diffusion of a multitude of unsatisfactory materials for use, which it is plain are derogatory to the growth of professional character.

Louis Jack,
*President of International Dental Publishing Company.*

